# NanoChest-Net: A Simple Convolutional Network for Radiological Studies Classification

**DOI:** 10.3390/diagnostics11050775

**Published:** 2021-04-26

**Authors:** Juan Eduardo Luján-García, Yenny Villuendas-Rey, Itzamá López-Yáñez, Oscar Camacho-Nieto, Cornelio Yáñez-Márquez

**Affiliations:** 1 Centro de Investigación en Computación, Instituto Politécnico Nacional, Mexico City 07700, Mexico; jeduardolujan5@gmail.com; 2 Centro de Innovación y Desarrollo Tecnológico en Cómputo, Instituto Politécnico Nacional, Mexico City 07738, Mexico

**Keywords:** X-ray classification, radiological images, convolutional neural network, deep learning, computer vision

## Abstract

The new coronavirus disease (COVID-19), pneumonia, tuberculosis, and breast cancer have one thing in common: these diseases can be diagnosed using radiological studies such as X-rays images. With radiological studies and technology, computer-aided diagnosis (CAD) results in a very useful technique to analyze and detect abnormalities using the images generated by X-ray machines. Some deep-learning techniques such as a convolutional neural network (CNN) can help physicians to obtain an effective pre-diagnosis. However, popular CNNs are enormous models and need a huge amount of data to obtain good results. In this paper, we introduce NanoChest-net, which is a small but effective CNN model that can be used to classify among different diseases using images from radiological studies. NanoChest-net proves to be effective in classifying among different diseases such as tuberculosis, pneumonia, and COVID-19. In two of the five datasets used in the experiments, NanoChest-net obtained the best results, while on the remaining datasets our model proved to be as good as baseline models from the state of the art such as the ResNet50, Xception, and DenseNet121. In addition, NanoChest-net is useful to classify radiological studies on the same level as state-of-the-art algorithms with the advantage that it does not require a large number of operations.

## 1. Introduction

The new coronavirus disease (COVID-19) has achieved historical records. Until 8 March 2021, the World Health Organization (WHO) had registered more than 116 million confirmed cases and over 2.5 million deaths [1]. COVID-19 is an infectious disease caused by SARS-CoV2 virus that affects severely the lungs of people infected, and the virus is easily propagated in the air and by contact. COVID-19 can cause complications and lead to development of pneumonia and other symptoms that can be confused with other diseases [1].

In addition, pneumonia is also an infectious disease that affects the lungs and can be caused by bacteria such as *Streptococcus pneumoniae* and *Haemophilus influenzae*, and viruses apart from the one that provokes COVID-19. It has been a major disease and cause of death for children and senior people around the world. According to the WHO, pneumonia causes 15% of all deaths of children under 5 years old [2]. Moreover, pneumonia killed 808,694 children in 2017.

On the other hand, tuberculosis disease, caused by *Mycobacterium tuberculosis* is also an infectious disease that causes antimicrobial resistance and death of tissue on different parts of the body, affecting principally the lungs. According to the WHO, tuberculosis is the top infectious killer around the world and causes around 1.5 million deaths every year. In 2019 alone, an estimated 10 million people fell ill with tuberculosis [3].

At the same time, according to the World Cancer Research Fund (WCRF) breast cancer is the most common cancer in women, and until 2018 it was the second most common cancer overall in the world, with over 2 million new cases in the same year [4]. Consequently, COVID-19, pneumonia, tuberculosis, and breast cancer have one thing in common: they can be diagnosed using radiological studies such as X-ray images. Lung infections can be detected by taking a chest X-ray of the patient; breast cancer can be detected by taking an X-ray image of the breast of a woman, called mammography [5]. In the case of COVID-19, radiological features include peripheral damage in one or both lungs, and a crazy paving pattern is commonly found in chest X-ray images from infected patients [1]. On the other hand, pneumonia not caused by COVID-19 causes pus and fluid in the lung, identifying radiopaque segments in the X-ray images without a specific pattern [6]. Tuberculosis presents radiolucent segments on the X-ray images due to solid necrosis in the center of the affected area (tubercles) [7]. As breast cancer concerns, mammography images show nodules and microcalcifications (radiopaque segments) near the mammary glands [8].

X-ray images usually are not as accurate as computed tomography (CT) and magnetic resonance imaging (MRI), but developing countries do not always have specialized equipment available to acquire CT and MRI. Therefore, X-ray images become a crucial tool to help physicians to diagnose diseases. With radiological studies and technology, computer-aided diagnosis (CAD) results in a very useful technique to analyze and detect abnormalities using the images generated by X-ray machines [9].

Prior to deep learning (DL) frameworks, medical image classification was based on traditional feature extraction and classification algorithms. For example, Livieris et al. [10] presented a framework that consisted in an ensemble of semi-supervised learning (SSL) algorithms to identify and classify lung abnormalities. In this work, authors used a tuberculosis dataset and different configurations of SSL such as self-training, co-training, and tri-training, obtaining accuracies under 74% for tuberculosis classification. Another popular work was presented by Minaee et al. [11] in which they manually extracted features from MRI to track the damage on patients with brain injuries. They used feature selection and linear regression as classification algorithms. Nowadays, CAD is mostly aided by computer vision (CV)-specialized algorithms from DL such as a convolutional neural network (CNN) [12]. CNNs are the most popular type of DL algorithms and the most used for medical image diagnosis. We can find several works from segmentation of lesions [13,14,15] to classification of different diseases [16,17,18,19].

As a major relevance for this work, we can find works that use radiological studies to classify among different diseases. Rajan et al. [20] presented a few-shot learning approach to classify among 14 chest diseases using X-ray images, and they proposed a solution to train a CNN with few data and solve the problem of acquiring a vast amount of medical imaging data.

As COVID-19 concerns, Sharma et al. [21] presented a CNN called CORONA-19 NET in which they used transfer learning to classify with a MobileNetV2 between normal and sick patients using a small dataset of 20 images. In addition, Zebin and Rezvy [22] used multiple pretrained CNNs as feature extractors to classify among patients. Moreover, Yu et al. [23] presented a framework that used four pretrained CNNs as baseline to classify among patients using CT scans, obtaining accuracies superior to 94%. Luján-García et al. [24] used an Xception CNN to classify among COVID-19 and pneumonia patients using a pretrained model on ImageNet. They showed that the Xception network was the fastest among several baselines. More recently, Yazdani et al. [25] presented a CNN with an attention mechanism to classify COVID-19 patients, obtaining a sensitivity of 90% using CT scans. Finally, Gupta et al. [26] presented a framework called InstaCovNet-19, which consists in using five pretrained baselines and stacking them to classify patients using X-ray images, and this obtained excellent results of almost 100% accuracy compared with other researches.

Pneumonia classification also plays an important role in radiological classification studies. Zhang et al. [27] presented a confidence-aware framework that uses a CNN as a feature extractor, a confidence module, and a prediction module achieving a sensitivity of 71.70%. Rahman et al. [28] presented a comparison between several baseline models to classify images of children infected with pneumonia, achieving up to 99% of sensitivity, and Luján-García et al. [29] used the same dataset but added a preprocessing technique and used a different pretrained baseline.

Recently, Rajpurkar et al. [30] presented a DL assistance tool to classify tuberculosis from patients with human immunodeficiency virus (HIV) using a CNN and a linear classifier to predict six clinical findings. On the other hand, Pasa et al. [31] presented a new small CNN to classify X-ray images from two small datasets, and they achieved good results despite the fact that no pretrained models were used. Moreover, using the same dataset as Pasa et al., Khatibi et al. [32] used an ensemble of CNNs to achieve classification accuracies up to 99.2%.

On the other hand, breast cancer has been improved using DL techniques. Shen et al. [33] used pretrained baselines on a large mammography dataset to classify among malign and benign mass and calcification, obtaining a sensitivity of 96%. Moreover, Agarwal [34] used a pretrained CNN to detect masses in mammography images, which achieved a better result, compared to Shen et al., with a sensitivity of 98% on the same dataset. Finally, Wu et al. [35] presented a custom ResNet-based CNN to classify over 1 million images from multiple views of patients with benign and malign masses, achieving an area under the curve score of 0.895.

Nonetheless, popular CNNs are enormous models and need a large amount of data for the purpose of being trained properly to get good results. Therefore, we aim to preset a small but effective CNN model that can be used to classify among different diseases using images from radiological studies.

## 2. Materials and Methods

In this section, datasets used for this research are described. In addition, we briefly introduce some of the CNN baseline models used for comparison purposes. Finally, metrics used for evaluating the algorithms are detailed.

### 2.1. Datasets

#### 2.1.1. Tuberculosis Dataset

The tuberculosis dataset is a collection of two sets of chest X-ray images from two different hospitals presented by the National Institute of Health of the United States [36]. The tuberculosis dataset is divided in two sets: the Montgomery County set and the Shenzhen set.

The Montgomery County set contains 138 frontal chest X-ray images, in which 80 of them are normal cases and 58 are from tuberculosis patients. Similarly, the Shenzhen set contains 662 frontal chest X-ray images, of which 326 are normal cases and 336 are tuberculosis patients.

#### 2.1.2. Pneumonia Children Dataset

The Pneumonia children dataset was published by Kermany et al. [37]. The dataset contains 5856 chest X-ray images of healthy and sick children up to five years old. All images are given as a training set, with 5232 images and an official test set of 624 images. From the 5232 images, 3883 are from patients infected with pneumonia. The remaining 1349 images are from healthy children. On the other hand, the test set is divided as follows: 390 images are from pneumonia-infected children, and 234 images are from healthy children.

#### 2.1.3. COVID-19 Dataset

Presented by Cohen et al. [38], The COVID-19 Image Data Collection was one of the first open available datasets that contained chest X-rays from patients infected with COVID-19. We initially used the dataset from November 2020, which contained 930 images from different diseases such as pneumonia, severe acute respiratory syndrome (SARS), and middle east respiratory syndrome (MERS), among others. At this time, only 478 images were from patients infected with COVID-19. These images were used to generate two different sets of images to perform experiments, explained in the next section.

#### 2.1.4. RSNA Pneumonia Challenge Dataset

The RSNA Pneumonia Challenge (RSNA-PC) is the only competition (from Kaggle.com) to classify and provide bounding boxes for damaged areas of the lung caused by pneumonia. The dataset contains 26,684 unique chest X-ray images of both normal (29%) and not normal/opacities (71%) for the training set, and 3000 images for the test set.

#### 2.1.5. BCDR Dataset

The Breast Cancer Digital Repository (BCDR), by Moura and Guevara [39], offers multiple datasets for both digital and scanned mammography in which principal classes are malign and benign tumors. For this work, we have used only the two datasets of digital mammography.

The BCDR-D01 contains full-field digital mammography and is composed of 79 biopsy-proven lesions of 64 women, rendering 143 segmentations for 80 unique images of patients with benign tumors, and 57 patients with malign tumors.

The BCDR-D02 contains full-field digital mammography and is composed of 230 biopsy-proven lesions of 162 women, rendering 455 segmentations for 359 unique images of patients with benign tumors, and 48 patients with malign tumors.

### 2.2. CNN Models from the State of the Art

Back in 2015, since the formal introduction of Deep Learning [40], the research community has dedicated a lot of attention and effort on developing DL algorithms for different purposes, such as image recognition, CV, CAD systems, and natural language processing, among others. Due to the capacity of extracting features withing the algorithm itself from different kind of signals (including images), CNNs have achieved magnificent results. Nowadays, a huge number of CNN models exist and are used for distinct purposes. As a result, we can find custom models [16,19,31] and the ones that use key baselines for classification of different diseases [17,22,24,29,41,42,43].

For this research, we have compared the proposed method with the most popular CNNs used for computer vision tasks such as the ResNet50 [44], the Xception network [45], and the DenseNet121 [46].

### 2.3. Metrics

**Figure 1:**
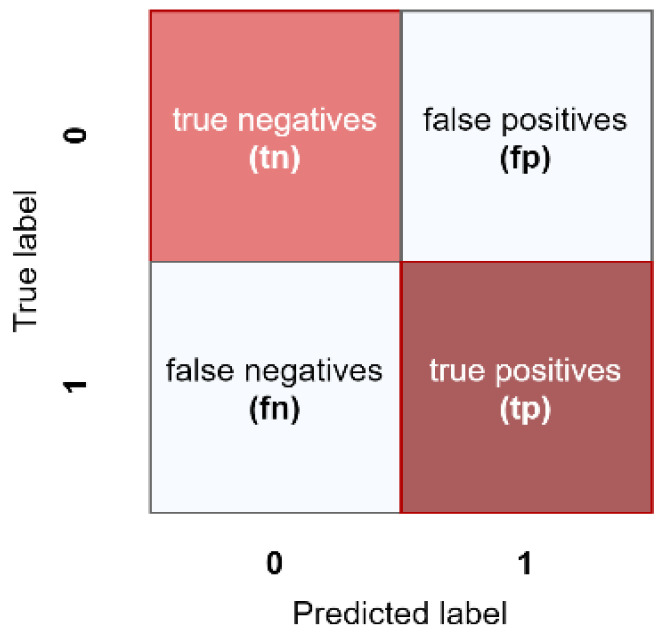
Confusion matrix for binary classification.

In a binary classification problem, we can measure the performance according to the examples correctly classified that belong to each class as true positives (tp) and true negatives (tn), and we take into account the mistakes or errors when classifying instances such as the false positives (fp) and false negatives (fn). Normally, tp, tn, fp, and fn are shown in tabular form as a confusion matrix (Figure 1).

From the confusion matrix, we can compute a variety of metrics. Accuracy is commonly used when we have a classification task among two or more different classes. Moreover, we can also compute other metrics such as precision, sensitivity, specificity, F1-Score, and the area under the ROC curve (AUC) [47]. Following, we can find the definition of the metrics used in this work (Equations (1)–(5)).

(1) $$Accuracy=\frac{tp+tn}{tp+fn+fp+tn}$$

(2) $$Precision=\frac{tp}{tp+fp}$$

(3) $$Sensitivity=\frac{tp}{tp+fn}$$

(4) $$Specificity=\frac{tn}{fp+tn}$$

(5) $$F1-Score=\frac{2\;tp}{2\;tp+fp+tn}=2\frac{\;precision\times recall}{precision+recall}.$$

In general, accuracy not always represents an unbiased performance measurement due to different imbalances within the instances of a dataset. Therefore, precision, sensitivity, specificity, F1-Score, and AUC are always helpful to measure the performance of a model. For this work, an AUC using thresholds was computed.

## 3. Proposal

In this section, a detailed description of the proposed custom CNN model is given. Moreover, final datasets and their partitions are explained. On the other hand, preprocessing and data augmentation techniques are described. Finally, hyperparameters used to train the models are mentioned.

### 3.1. DL Model

**Figure 2:**
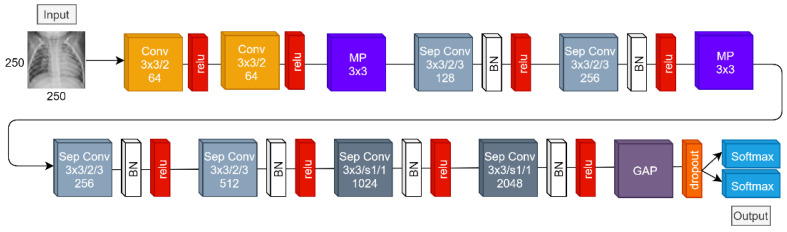
Block diagram of NanoChest-net.

Inspired by the Separable Convolutions from the Xception network, we have designed the NanoChest-net to classify between images from radiological studies, such as X-ray images. The complete block diagram of our CNN model is shown in Figure 2. A complete specification of each layer of the CNN is described in Table 1.

We have used the depth multiplier of Separable Convolution layers to increment the number of output channels on each layer. In addition, we have used a dilation rate of 2 to increment the size of the spatial perception field on each layer. As a result, Table 1 shows us the total number of layers of our proposal, which is 28. If we count the set of a convolutional layer, the batch normalization layer, and its activation as a complete layer (as commonly used in the literature), then our proposal is composed of a very small number of 14 layers. Moreover, if we only focus on weighted layers, then our proposal is as small as 10 layers in depth. In comparison, baseline models such as VGG-16, which comes next in layer size, contain 19 weighted layers and have an average of 136.4 million parameters [48]. Therefore, our proposal has the advantage of halving the depth according to weighted layers, and it has 40 times fewer parameters with only 3.4 million.

As consequence, the aforementioned reasons are motive to call this small model as NanoChest-net due to the minimal number of layers on the CNN model and its application to radiological studies, primarily of the chest.

### 3.2. Datasets Splitting and Validation Method

#### 3.2.1. Splitting and Final Datasets

We have maintained the original examples for Shenzhen, Montgomery, Pneumonia children, BCDR-D01, and BCDR-D02. Nonetheless, we have generated two new subsets using the COVID-19 dataset and the RSNA-PC dataset. We took the 478 COVID-19 images from the COVID-19 dataset and 478 images of healthy patients of the RSNA-PC to generate the COVID-NORMAL dataset. Moreover, we took the same 478 images from COVID-19 dataset, but now 478 images of pneumonia-sick patients from the RSNA-PC to generate the COVID-PNEUMONIA dataset. Table 2 shows the final datasets used for this research.

#### 3.2.2. Validation Method

**Figure 3:**
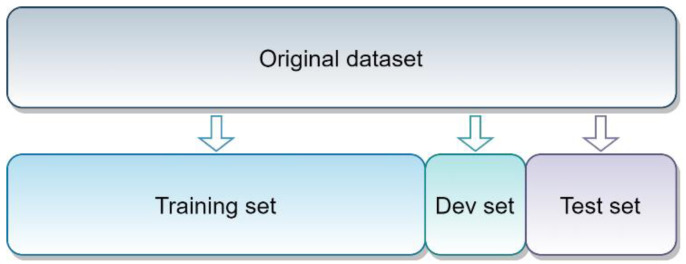
Hold-out validation method.

Hold-out validation was performed in order to obtain the training, development (Dev), and test set for each dataset. Hold-out validation consists of randomly dividing the original number of images on the training, Dev, and test set. Figure 3 shows the behavior of the hold-out validation method.

A hold-out 70-10-20 was used over each dataset, except for the Pneumonia children, in which an official test set was established by the authors. Therefore, partitions for each dataset are as follows (Table 3).

### 3.3. Preprocessing and Data Augmentation

All images were normalized before feeding the CNN models. In addition, all datasets were resized to 500 × 500 pixels to avoid resizing each image from its original size to the input size of each CNN model on each step of the training. Moreover, we fed the models with the original input size. Then, the input size of each model is as follows (Table 4).

#### Tuberculosis Montgomery County Dataset

**Figure 4:**
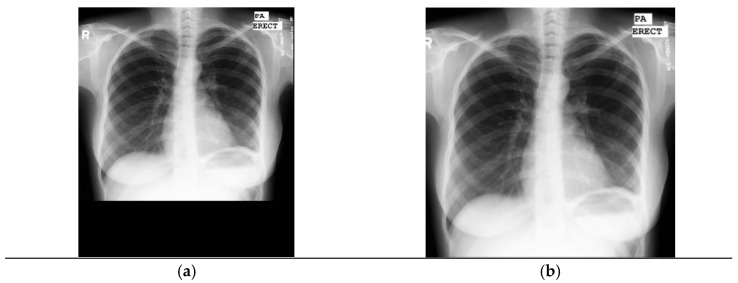
Center region cropping applied to the Montgomery County dataset. (**a**) Shows the original image with black bands; (**b**) shows the preprocessed image.

For the Montgomery County dataset, first we cropped the central region of the images with the intention of deleting black bars from the original images (Figure 4). We followed the algorithm by Pasa et al. [31]. Then, we applied preprocessing and data augmentation techniques.

On the other hand, data augmentation techniques were applied to each dataset aiming to obtain a better generalization of the models. For tuberculosis datasets, pneumonia dataset, and COVID-19 dataset we applied horizontal flip, magnification in a range of 0.90 to 1.2, random width and height shift with a factor of 0.20, random rotation of 20 degrees, and brightness changes in a factor range of 0.80 to 1.05. In the case of the BCDR datasets, we changed the random rotation to 30 degrees and added vertical flip.

### 3.4. Hyperparameter Tuning

We conducted the same experiments using the state-of-the-art CNNs and our proposed method. Equivalent hyperparameters were used through all models, except for the input size. We used the original input size for each model, as mentioned in Section 4.3. We trained all models using a logistic layer of two units with Sigmoid activation to get the probability of having each of two classes per dataset. Binary cross-entropy was used as a cost function (computed as in [29]) and Adam [49] as optimization algorithm with parameters $\beta_{1}=0.9,\;\beta_{2}=0.999$ (recommended values from original paper). In addition, we performed several experiments with different optimizers to see the impact in the training of our proposal as seen in Table 5 (best results are highlighted in **bold**).

From Table 5, we can see that the stochastic gradient descent (SGD) [50] did not obtain good results in any dataset. On the other hand, Adam obtained the best scores 25 times, and RMSProp (introduced by Hinton in 2012) obtained the best scores 17 times. Adam was selected due to the fact it is a combination of SGD (using momentum) and RMSProp (squared gradients). As a result, we also performed experiments to see the impact of changing the learning rate for training using Adam optimizer as seen on Table 6 (best results are highlighted in **bold)**.

From previous results (Table 6) we can see that using a learning rate of 0.001 provided the best scores 16 times. Nonetheless, using a learning of 0.0005, the best scores were obtained 23 times. A learning rate of 0.0005 was selected because it showed better performance than a larger one. Moreover, a small learning rate on the order of 10–4 benefits all models when training on small datasets.

As a result, a learning rate of 0.0005 was selected to perform all experiments with all datasets. In the same way, the Adam algorithm, which is a combination of SGD and RMSProp, was selected as an optimization algorithm. Moreover, the number of epochs was selected according to the size of each dataset and our technological capabilities. In addition, the batch size was selected considering the size of each dataset and its partitions. Learning rate, epochs, and batch size configurations are shown in Table 7.

We applied weights to BCDR-D01 and BCDR-D02 datasets to combat class imbalance. We used 0.8636 and 1.1875 for benign and malign on BCDR-D01, and 0.568 and 4.1765 for benign and malign on BCDR-D02.

## 4. Results

In this section, the experimental framework is described. In addition, performance and comparison between models are presented. Furthermore, statistical analysis is presented, considering metrics and time measurements.

### 4.1. Experimental Framework

Experiments for this research were conducted on a PC with AMD Ryzen 3700x processor; 16 GB of RAM; 512 SSD + 2 TB of storage; GPU Nvidia RTX 2070 Super with 8 GB GDDR5; Python 3.7.9 was used as programming language; TensorFlow 2.1.0 with Keras as high-level DL framework; sci-kit learn 0.23.2 [51] as machine learning (ML) library; and OpenCV 3.4.2 [52] as main image processing library. Moreover, we set a fixed seed for TensorFlow, Python random generator, and NumPy library to get the repeatability of the experiments.

In addition, we want to clarify that all baseline CNN models have been randomly initialized with the intention of making a fair comparison with our proposal. Neither transfer learning nor finetuning were performed in these tests. Apart from state-of-the-art works [13,14,15,16,17,18,19,20,21,22,23,24,25,26,27,28,29,30,32,33,34,35] pretrained in several medical image datasets and ImageNet, our proposal received no extra training and only took the training partition of each presented dataset. Our source code is available on https://github.com/zotrick/NanoChest-net.

### 4.2. Test Sets Results

We trained all baseline models and the proposed NanoChest-net using the same hyperparameters (apart from the input size) on all the datasets specified in Section 2 and computed the performance metrics. The results over the respective test set for each dataset can be found in Table 8 (best results are highlighted in **bold**).

As seen in Table 8. The proposed model behaved similar to the baseline ones. A detailed discussion will be given in the next section.

### 4.3. Training Time Results

Apart from evaluating the metrics, we also measured the time taken for each model at training time. We measured the total time taken by each model, the average time per epoch, the time of each model to process a single example, and the time taken in achieving the best result through all the training epochs. Therefore, results can be found in Table 9 (best results are highlighted in **bold**).

Despite the fact our proposal did not seem be faster among the CNN models, we will perform further analyses in the next section.

### 4.4. Size of the Models

With the final structure of each CNN model, and after training them, we measured the total number of parameters of each one. In addition, as the best-performance model for all CNNs was saved, we measured the size of storage of each model. Results can be found in Table 10 (smaller number of parameters and size are highlighted in **bold**).

### 4.5. Statistical Analysis

We performed the Friedman test [53] over computed metrics for each model on each dataset (Table 11). The Friedman test tells with a 95% confidence if a significant statistical difference exists between five or more instances ranked in order of significance. If *p* < 0.05 is found, then significant differences exist. We arranged the results of the Friedman test in Table 11 (best results are highlighted in **bold**).

From Table 11, we can observe that *p*-values were greater than 0.05 (confidence level of 95%). Therefore, the null hypothesis for equality on the performance of compared algorithms was not rejected.

As for time per example, statistical analysis showed that the Friedman test obtained a *p*-value of 0.001213, being the ResNet50 the best of the ranking. As a result, the null hypothesis was rejected. However, after applying the Holm test [54], we obtained the results as shown in Table 12.

From Table 12, we can observe that the Holm test rejected the hypothesis with unadjusted *p*-values smaller than 0.001213. Therefore, neither DenseNet121 nor NanoChest-net was rejected. On the contrary, only the Xception network was rejected, showing significant differences (inferior performance) compared with the other algorithms. Consequently, we will perform further analyses in the next section.

## 5. Discussion

In this section, advantages of the proposal are highlighted. An evaluation of the classification results on the different datasets is performed, as well as the time analysis of the training.

From the classification results (Table 8), our proposal obtained good results through all the datasets. On the Montgomery County dataset, our model obtained the best results on accuracy, sensitivity, F1-Score, and AUC, with scores of 0.931, 1.000, 0.929, and 0.928, respectively. For the Shenzhen dataset, our model obtained the second-best scores on accuracy (0.828), sensitivity (0.897), F1-Score (0.841), and AUC (0.928) only behind the Xception network. On the Pneumonia children dataset, our method achieved the best results among all CNN models, with an accuracy of 0.931, precision of 0.904, sensitivity of 0.995, specificity of 0.825, F1-Score of 0.947, and an AUC score of 0.992. Again, for the COVID-NORMAL dataset, our proposal achieved the best scores of all models with an accuracy of 0.933, precision score of 0.912, sensitivity score of 0.959, specificity score of 0.907, F1-Score of 0.935, and an AUC score of 0.970. On the other hand, for the COVID-PNEUMONIA dataset, the NanoChest-net obtained the best results for precision (0.860), specificity (0.878), and AUC (0.919), and the remaining metrics had good results, only behind the Xception network. Nevertheless, for the BCDR-D01 dataset, we fell behind to third place behind DenseNet121 and Xception networks, except for sensitivity (best result). We obtained the following scores: accuracy of 0.621, precision of 0.500, sensitivity of 0.818, specificity of 0.500, F1-Score of 0.621, and AUC of 0.702. Finally, for the BCDR-D02 dataset we obtained the best results for sensitivity (0.556) and F1-Score (0.278). For the remaining metrics, we obtained second place only behind the DenseNet121 with an accuracy of 0.687, precision of 0.185, specificity of 0.703, and an AUC of 0.664. Finally, from the statistical analysis for metrics (Table 9), the Friedman test did not show evidence to reject the hypothesis. Therefore, there were no statistically significant differences among the models. However, the scores from the test placed our proposal at the top of the ranking on each metric, showing a superior behavior compared to the state-of-the-art baseline models. In addition, if we used a confidence level of 90%, then there will exist differences on F1-Score and AUC in favor of NanoChest-net due to its first position in the ranking.

On the other hand, time measurement results (Table 9) showed that in the Montgomery County and BCDR-D01 our proposal obtained the lowest values for total training time, epoch average time, and time per example. For Shenzhen, Pneumonia children, COVID-NORMAL, COVID-PNEUMONIA, and BCDR-D02, our method was always in third place, behind the ResNet50 and DenseNet121 networks. Nonetheless, from the Friedman test and Holm results (Table 11 and Table 12) we can observe that there were no significant differences among ResNet50, DenseNet121, and our method. The Xception network was the only worse model considering the time taken per example.

At the same time, our proposal took an important step further on a crucial aspect. Apart from showing decent classification and time results, our method was significantly smaller in parameter count and storage size. From Table 10, we can observe that our method had less than half the parameters and size compared to DenseNet121, and 6 to 7 times fewer parameters and smaller size when compared to Xception and ResNet50 models. Consequently, our model could be used in computers, embedded devices, and mobile devices with limited storage, memory, and computation capabilities.

## 6. Conclusions

In this paper, we have introduced a new, full custom, and small convolutional neural network model called NanoChest-net. Our proposed model is used to classify medical images from radiological studies such as X-rays from the chest and mammography from the breasts of women. As a result, our model proves to be effective in classifying among different diseases like tuberculosis, pneumonia, and COVID-19. Moreover, NanoChest-net obtained the best results on both the Pneumonia children and COVID-NORMAL datasets. On the remaining datasets, our model proved to be as good as baseline models from the state of the art such as the ResNet50, Xception, and DenseNet121, finding no statistically relevant differences among models, neither in performance nor training time. On the contrary, we can find an abrupt difference on the number of parameters and storage size of our model, being two to seven times smaller compared with baseline models. In short, the NanoChest-net model is useful to classify radiological studies on the same level as state-of-the-art algorithms and without computing large numbers of operations and occupying more than 40 MB of storage, making our proposal suitable for embedded and mobile devices.

As future work we plan to further study the correlation of radiological features between pneumonia caused by COVID-19 and other viruses or bacteria. In addition, we plan to train the NanoChest-net on ImageNet and perform a comparison with state-of-the-art frameworks for medical image classification.

## Figures and Tables

**Table 1:** Layer specification of NanoChest-net.

Layer Type	Specifications
Input	Size = (250, 250, 3)
Convolution	Number of filters = 64 kernel size = (3, 3) dilatation rate = 2 padding = valid
Relu	Nonlinearity relu
Convolution	Number of filters = 64 kernel size = (3, 3) dilatation rate = 2 padding = valid
Relu	Nonlinearity relu
Max Pooling	Pool size = (3, 3)
Separable Convolution	Number of filters = 128 kernel size = (3, 3) dilatation rate = 2 depth multiplier = 3 padding = valid
Batch Normalization	Normalization
Relu	Nonlinearity relu
Separable Convolution	Number of filters = 256 kernel size = (3, 3) dilatation rate = 2 depth multiplier = 3 padding = valid
Batch Normalization	Normalization
Relu	Nonlinearity relu
Max Pooling	Pool size = (3, 3)
Separable Convolution	Number of filters = 256 kernel size = (3, 3) dilatation rate = 2 depth multiplier = 3 padding = valid
Batch Normalization	Normalization
Relu	Nonlinearity relu
Separable Convolution	Number of filters = 512 kernel size = (3, 3) dilatation rate = 2 depth multiplier = 3 padding = valid
Batch Normalization	Normalization
Relu	Nonlinearity relu
Separable Convolution	Number of filters = 1024 kernel size = (3, 3) padding = same
Batch Normalization	Normalization
Relu	Nonlinearity relu
Separable Convolution	Number of filters = 2048 kernel size = (3, 3) padding = same
Batch Normalization	Normalization
Relu	Nonlinearity relu
Global Average Pooling	Global Pooling
Dropout	Keeping rate = 0.25
Logistic-Output	Units = 2 activation = Softmax

**Table 2:** Number of images of each class from each dataset.

Dataset	Classes	Images per Class	Official Test Set
Montgomery County	{NORMAL, TUBERCULOSIS}	80, 58	-
Shenzhen	{NORMAL, TUBERCULOSIS}	326, 336	-
Pneumonia children	{NORMAL, PNEUMONIA}	1349, 3883	234, 390 [37]
COVID-NORMAL	{COVID, NORMAL}	478, 478	-
COVID-PNEUMONIA	{COVID, PNEUMONIA}	478, 478	-
BCDR-D01	{BENIGN, MALIGN}	80, 57	-
BCDR-D02	{BENIGN, MALIGN}	359, 48	-

**Table 3:** Partitions for each dataset.

Dataset	Partition	Class 1	Class 2
Montgomery County	Training set	56	40
	Dev set	8	5
	Test set	16	13
Shenzhen	Training set	228	235
	Dev set	32	33
	Test set	66	68
Pneumonia children	Training set	1214	3494
	Dev set	135	389
	Test set	234	390
COVID-NORMAL	Training set	334	334
	Dev set	47	47
	Test set	97	97
COVID-PNEUMONIA	Training set	334	334
	Dev set	47	47
	Test set	97	97
BCDR-D01	Training set	56	40
	Dev set	8	6
	Test set	16	11
BCDR-D02	Training set	251	34
	Dev set	36	5
	Test set	72	9

**Table 4:** Input size for each CNN model.

Model	Input Size
ResNet50	224 × 224 × 3
Xception	299 × 299 × 3
DenseNet121	224 × 224 × 3
NanoChest-net	250 × 250 × 3

**Table 5:** Results using different optimizers for NanoChest-net.

Dataset	Optimizer	Accuracy	Precision	Sensitivity	Specificity	F1	AUC
Montgomery County	SGD	0.552	0.000	0.000	1.000	0.000	0.587
	RMSProp	0.862	0.765	1.000	0.750	0.867	0.981
	Adam	**0.931**	**0.867**	**1.000**	**0.875**	**0.929**	**0.928**
Shenzhen	SGD	0.739	0.739	0.750	0.727	0.745	0.861
	RMSProp	**0.881**	**0.906**	0.853	**0.909**	**0.879**	**0.932**
	Adam	0.828	0.792	**0.897**	0.758	0.841	0.928
Pneumonia children	SGD	0.894	0.860	0.992	0.731	0.921	0.984
	RMSProp	0.920	0.886	**1.000**	0.786	0.940	**0.994**
	Adam	**0.931**	**0.904**	0.995	**0.825**	**0.947**	0.992
COVID-NORMAL	SGD	0.732	0.696	0.825	0.639	0.755	0.844
	RMSProp	0.871	0.860	0.887	0.856	0.873	0.930
	Adam	**0.933**	**0.912**	**0.959**	**0.907**	**0.935**	**0.970**
COVID-PNEUMONIA	SGD	0.694	0.679	0.735	0.653	0.706	0.787
	RMSProp	0.786	0.780	**0.796**	0.776	0.788	0.881
	Adam	**0.816**	**0.860**	0.755	**0.878**	**0.804**	**0.919**
BCDR-D01	SGD	0.483	0.250	0.182	0.667	0.211	0.379
	RMSProp	**0.724**	**0.636**	0.636	**0.778**	**0.636**	**0.768**
	Adam	0.621	0.500	**0.818**	0.500	0.621	0.702
BCDR-D02	SGD	0.639	0.161	0.556	0.649	0.250	0.679
	RMSProp	0.614	**0.189**	**0.778**	0.595	**0.304**	**0.707**
	Adam	**0.687**	0.185	0.556	**0.703**	0.278	0.664

**Table 6:** Results using Adam optimizer and different learning rates for NanoChest-net.

Dataset	Learning Rate	Accuracy	Precision	Sensitivity	Specificity	F1	AUC
Montgomery County	0.001	0.793	0.684	1.000	0.625	0.813	**1.000**
	0.0005	**0.931**	**0.867**	**1.000**	**0.875**	**0.929**	0.928
Shenzhen	0.001	**0.858**	**0.866**	0.853	**0.864**	**0.859**	**0.937**
	0.0005	0.828	0.792	**0.897**	0.758	0.841	0.928
Pneumonia children	0.001	0.931	**0.906**	0.992	**0.829**	0.947	0.992
	0.0005	0.931	0.904	**0.995**	0.825	0.947	0.992
COVID-NORMAL	0.001	0.861	0.830	0.907	0.814	0.867	0.927
	0.0005	**0.933**	**0.912**	**0.959**	**0.907**	**0.935**	**0.970**
COVID-PNEUMONIA	0.001	**0.847**	**0.886**	**0.796**	**0.898**	**0.839**	0.869
	0.0005	0.816	0.860	0.755	0.878	0.804	**0.919**
BCDR-D01	0.001	0.690	0.583	0.636	**0.722**	0.609	0.657
	0.0005	0.621	0.500	**0.818**	0.500	**0.621**	**0.702**
BCDR-D02	0.001	0.458	0.109	0.556	0.446	0.182	0.545
	0.0005	**0.687**	**0.185**	**0.556**	**0.703**	**0.278**	**0.664**

**Table 7:** Hyperparameters for each dataset.

Dataset	Learning Rate	Epochs	Batch Size
Montgomery County	0.0005	200	4
Shenzhen	0.0005	200	8
Pneumonia children	0.0005	100	16
COVID-NORMAL	0.0005	200	16
COVID-PNEUMONIA	0.0005	200	16
BCDR-D01	0.0005	200	4
BCDR-D02	0.0005	200	8

**Table 8:** Metrics of CNN models over all datasets.

Dataset	Model	Accuracy	Precision	Sensitivity	Specificity	F1	AUC
Montgomery County	ResNet50	0.862	**1.000**	0.692	**1.000**	0.818	0.885
	Xception	0.690	0.611	0.846	0.563	0.710	0.851
	DenseNet121	0.793	0.818	0.692	0.875	0.750	0.755
	NanoChest-net	0.931	0.867	**1.000**	0.875	**0.929**	**0.928**
Shenzhen	ResNet50	0.813	**0.864**	0.750	**0.879**	0.803	0.871
	Xception	**0.851**	0.800	**0.941**	0.758	**0.865**	**0.937**
	DenseNet121	0.776	0.788	0.765	0.788	0.776	0.883
	NanoChest-net	0.828	0.792	0.897	0.758	0.841	0.928
Pneumonia children	ResNet50	0.921	0.892	**0.995**	0.799	0.941	0.990
	Xception	0.917	0.886	**0.995**	0.786	0.937	0.992
	DenseNet121	0.921	0.894	0.992	0.803	0.940	0.989
	NanoChest-net	**0.931**	**0.904**	**0.995**	**0.825**	**0.947**	**0.992**
COVID-NORMAL	ResNet50	0.845	0.802	0.918	0.773	0.856	0.953
	Xception	0.887	0.871	0.907	0.866	0.889	0.960
	DenseNet121	0.866	0.890	0.835	0.897	0.862	0.924
	NanoChest-net	**0.933**	**0.912**	**0.959**	**0.907**	**0.935**	**0.970**
COVID-PNEUMONIA	ResNet50	0.796	0.796	0.796	0.796	0.796	0.843
	Xception	**0.837**	0.824	**0.857**	0.816	**0.840**	0.872
	DenseNet121	0.776	0.755	0.816	0.735	0.784	0.857
	NanoChest-net	0.816	**0.860**	0.755	**0.878**	0.804	**0.919**
BCDR-D01	ResNet50	0.586	0.474	**0.818**	0.444	0.600	0.662
	Xception	0.655	0.571	0.364	**0.833**	0.444	**0.732**
	DenseNet121	**0.759**	**0.667**	0.727	0.778	**0.696**	0.854
	NanoChest-net	0.621	0.500	**0.818**	0.500	0.621	0.702
BCDR-D02	ResNet50	0.590	0.143	**0.556**	0.595	0.227	0.659
	Xception	0.627	0.156	**0.556**	0.635	0.244	0.565
	DenseNet121	**0.735**	**0.190**	0.444	**0.770**	0.267	**0.673**
	NanoChest-net	0.687	0.185	**0.556**	0.703	**0.278**	0.664

**Table 9:** Time measurements of CNN models over all datasets.

Dataset	Model	Total Training Time (s)	Epoch Avg Time (s)	Time per Example (s)	Convergence Time (s)
Montgomery County	ResNet50	251.8598	1.2593	0.0131	166.2275
	Xception	490.9686	2.4548	0.0256	198.8423
	DenseNet121	268.7426	1.3437	0.0140	**143.7773**
	NanoChest-net	**227.5804**	**1.1379**	**0.0119**	216.2014
Shenzhen	ResNet50	**955.6777**	**4.7784**	**0.0105**	793.2125
	Xception	2112.3239	10.5616	0.0232	1193.4630
	DenseNet121	997.1672	4.9858	0.0109	623.2295
	NanoChest-net	1071.1402	5.3557	0.0117	**599.8385**
Pneumonia children	ResNet50	**4649.3123**	**46.4931**	**0.0099**	4416.8467
	Xception	9404.9841	94.0498	0.0200	7241.8378
	DenseNet121	4898.9102	48.9891	0.0104	4115.0845
	NanoChest-net	5474.7824	54.7478	0.0116	**3941.8433**
COVID-NORMAL	ResNet50	**1317.2370**	**6.5862**	**0.0100**	1172.3409
	Xception	2691.6571	13.4583	0.0205	**753.6640**
	DenseNet121	1384.6404	6.9232	0.0106	851.5538
	NanoChest-net	1518.7023	7.5935	0.0116	1260.5229
COVID-PNEUMONIA	ResNet50	**1387.4420**	**6.9372**	**0.0106**	1200.1374
	Xception	2796.1585	13.9808	0.0213	503.3085
	DenseNet121	1423.2266	7.1161	0.0108	1095.8845
	NanoChest-net	1581.9784	7.9099	0.0121	**450.8638**
BCDR-D01	ResNet50	245.4158	1.2271	0.0133	**158.2932**
	Xception	472.3512	2.3618	0.0257	340.0929
	DenseNet121	271.2206	1.3561	0.0147	269.8645
	NanoChest-net	**225.8349**	**1.1292**	**0.0123**	195.3472
BCDR-D02	ResNet50	**574.5628**	**2.8728**	**0.0103**	255.6805
	Xception	1239.4353	6.1972	0.0221	1171.2663
	DenseNet121	594.5677	2.9728	0.0106	**335.9307**
	NanoChest-net	630.9567	3.1548	0.0113	498.4558

**Table 10:** Size comparison of the CNN models.

Model	Total Parameters	Size (MB)
ResNet50	23,591,810	270
Xception	20,865,578	239
DenseNet121	7,039,554	81.8
**NanoChest-net**	**3,393,986**	**38.9**

**Table 11:** Friedman test over each metric for all models.

Model	Accuracy	Precision	Sensitivity	Specificity	F1-Score	AUC
	**Friedman Test**					
	0.183672	0.418818	0.170754	0.440227	0.085801	0.087436
	**Ranking**					
**NanoChest-net**	**1.71**	**1.8571**	**1.9286**	**2**	**1.4286**	**1.6429**
Xception	2.42	2.8571	2.1429	2.9286	2.7143	2.2143
DenseNet121	2.64	2.4286	3.3571	2.2143	2.8571	2.8571
ResNet50	3.21	2.8571	2.5714	2.8571	3	3.2857

**Table 12:** Adjusted p-values for time per example obtained through the post hoc method (Friedman).

i	Algorithm	Unadjusted *p*
1	Xception	0.000084
2	NanoChest-net	0.09769
3	DenseNet121	0.147299

## Data Availability

Tuberculosis datasets: available from National Institute of Health at https://lhncbc.nlm.nih.gov/LHC-publications/pubs/TuberculosisChestXrayImageDataSets.html (accessed on 25 October 2020); Pneumonia children dataset: available at https://data.mendeley.com/datasets/rscbjbr9sj/3 (accessed on 5 October 2020); COVID-19 dataset: available on GitHub repository at https://github.com/ieee8023/covid-chestxray-dataset (accessed on 1 November 2020). RSNA-PC dataset: available at https://www.kaggle.com/c/rsna-pneumonia-detection-challenge/ (accessed on 5 October 2020); BCDR datasets can be accessed by filling a form at https://bcdr.eu/ (accessed on 27 November 2020).
